# Effect of combat gear and body armor on ventilatory thresholds and muscle oxygenation during graded treadmill exercise in female and male soldiers

**DOI:** 10.3389/fphys.2026.1943048

**Published:** 2026-10-20

**Authors:** Jonas Larsson, Magnus Dencker, Ann Bremander, M. Charlotte Olsson

**Affiliations:** 1 Department of Medical Imaging and Physiology, Skåne University Hospital, Malmö, Sweden; 2 Department of Translational Medicine, Lund University, Lund, Sweden; 3 Department of Environmental and Biosciences, School of Business, Innovation and Sustainability, Halmstad University, Halmstad, Sweden; 4 Swedish Armed Forces, Eksjö, Sweden; 5 Department of Radiology, Medical Diagnostics, Highland Hospital, Eksjö, Sweden; 6 Department of Clinical Sciences, Lund, Section of Rheumatology, Lund University, Lund, Sweden; 7 Spenshult Research and Development Centre, Halmstad, Sweden

**Keywords:** body armor, cardiopulmonary exercise testing, load carriage, muscle oxygen saturation, near-infra read spectroscopy, sex differences, ventilatory transitions

## Abstract

**Introduction:**

This study aimed to determine how combat gear and body armor (CB) affect the relationship between treadmill setting and internal physiological responses at ventilatory transitions, and to characterize right vastus lateralis muscle oxygen saturation (SmO_2_) and exploratory early reoxygenation in female and male soldiers.

**Methods:**

Nine female and nine male soldiers completed randomized no-load (NL) and CB graded treadmill tests to volitional exhaustion. Heart rate and pulmonary gas exchange were measured, and right vastus lateralis SmO_2_ was assessed using near-infrared spectroscopy. VT1 was identified using the V-slope method and VT2 using ventilatory-equivalent criteria. SmO_2_ outcomes were referenced to a 30-s standing value.

**Results:**

VT1 was identified at a lower treadmill stage in CB than in NL in men (5.2 ± 0.8 vs. 6.4 ± 1.7; p = 0.02) and women (5.1 ± 0.6 vs. 7.1 ± 1.5; p <0.01). Absolute V̇O_2_ at VT1 changed little, whereas HR was higher in CB in men (p <0.01) and women (p = 0.04). VT2 was also identified at a lower stage in CB in both groups (p <0.01). Baseline-referenced right vastus lateralis desaturation amplitude during CB was greater in men than women (39.0% ± 23.9% vs. 17.1 ± 14.5%; p <0.01), although adipose-tissue thickness at the sensor site was not measured. No statistically significant effects were detected for the early post-exercise indices.

**Discussion:**

CB altered the treadmill setting at which ventilatory transitions were observed and increased cardiovascular demand at VT1 without essentially changing absolute V̇O_2_ at VT1. This indicates a changed relationship between treadmill task, transported mass, and internal physiological response rather than demonstrated displacement of the underlying metabolic threshold. The site-specific SmO_2_ and early reoxygenation findings were exploratory, and between-sex interpretation was limited by the small sample and unmeasured adipose-tissue thickness.

## Introduction

Combat gear and body armor (CB) protect soldiers but add mass and bulk that increase the physiological cost of locomotion and may constrain ventilation or movement during demanding tasks. Load carriage has been extensively studied in relation to oxygen uptake, ventilation, and heart rate, and reviews consistently describe greater cardiorespiratory strain when external mass is transported (Drain et al., 2016; Boffey et al., 2019; Faghy et al., 2022). However, treadmill speed or grade, total transported mass, and the resulting internal physiological response are distinct constructs. A lower treadmill setting in a loaded condition does not necessarily indicate displacement of an underlying metabolic threshold because the loaded and unloaded tasks differ in total transported mass.

Ventilatory thresholds are non-invasive transition markers that are associated with changes between exercise-intensity domains, although they are not necessarily identical to the corresponding metabolic-domain boundaries (Galán-Rioja et al., 2020; Keir et al., 2024). The first ventilatory threshold (VT1) is commonly associated with the transition from moderate to heavy exercise, whereas the second ventilatory threshold or respiratory compensation point (VT2/RCP) is associated with the transition toward severe exercise. Cardiopulmonary testing during loaded conditions may reproduce similar oxygen uptake and heart rate (HR) responses at these transitions despite different treadmill settings; a recent comparison of a vest-loaded incline protocol with an unloaded speed-ramp protocol reported practically comparable V̇O_2max_ and submaximal threshold responses (Dössegger et al., 2026). The physiologically relevant question is therefore whether CB changes the relationship between the task setting and internal responses such as V̇O_2_ and HR at the ventilatory transitions, rather than whether a lower stage alone represents a shifted metabolic threshold.

Local muscle oxygenation may provide information that complements pulmonary gas exchange. Near-infrared spectroscopy (NIRS) estimates skeletal-muscle oxygen saturation (SmO_2_) and reflects the local balance between oxygen delivery and utilization in the sampled tissue (Bhambhani et al., 1998). During incremental exercise, SmO_2_ commonly decreases as work rate increases, but the signal is site-specific and is affected by adipose-tissue thickness (Ferrari et al., 2004; Van der Zwaard et al., 2016). Early reoxygenation after exercise may also provide an exploratory local index of reperfusion, which may be relevant when military tasks require repeated efforts separated by short recovery periods (Nindl et al., 2018). It should not, however, be interpreted as recovery in general.

Potential sex-related differences are also relevant when evaluating load carriage. Men and women differ, on average, in body size, body composition (Epstein et al., 2013), and absolute aerobic capacity (Ogawa et al., 1992), although some differences in oxygen uptake are reduced when values are normalized to fat-free mass (Mitchell et al., 1992). Previous studies have reported sex-related differences in muscle oxygenation during exercise (Bhambhani et al., 1998; Espinosa-Ramirez et al., 2021), but findings are inconsistent and data from military populations wearing CB are scarce. Understanding whether female and male soldiers respond differently to loaded exercise may help inform physical testing, conditioning, and task preparation.

Accordingly, the primary aim was to determine how CB affected the treadmill stage and the internal physiological responses (V̇O_2_ and HR) observed at VT1 and VT2 during graded treadmill exercise. Secondary aims were to characterize baseline-referenced right vastus lateralis SmO_2_ responses and an exploratory index of early local reoxygenation, and to explore whether these responses differed between female and male soldiers. Peak aerobic responses, previously reported from the same experiment (Larsson et al., 2022), are included only to contextualize the threshold and relative-intensity analyses.

## Materials and methods

### Subjects

The participants and overall experimental protocol have been described previously in a report of cardiorespiratory responses to load carriage from the same experiment (Larsson et al., 2022). The present analysis focuses on additional outcomes related to ventilatory transitions, right vastus lateralis muscle oxygenation, and early post-exercise reoxygenation; peak outcomes are repeated only as contextual reference values. In brief, 18 soldiers (9 women) volunteered for the study: age 26.2 years ± 4.8 years (women) and 22.4 years ± 2.4 years (men); height 167.0 cm ± 6.0 cm (women) and 181.3 cm ± 5.2 cm (men). Their length of military service ranged from 1 to 5 years. The study was conducted in accordance with the Declaration of Helsinki (World Medical Association, 2013) and was approved by the Regional Ethical Review Board in Lund, Sweden (Dnr 2016/400). All participants were informed of the study procedures and provided written informed consent before participation.

### Study protocol

Body composition measurements were performed in a fasting state. Height was measured, and body mass, body fat, right-leg fat, and fat-free mass (FFM) were determined by bioelectrical impedance analysis (InBody 770, Biospace Co. Ltd., Seoul, Korea). A treadmill GXT adapted from the Bruce protocol (Bruce et al., 1963) was conducted indoors in a laboratory, at an ambient temperature of 21–25 °C and a relative humidity of 67–71%. Stage 1 comprised 5 min of marching at 5.4 km·h^−^¹ and 0% grade; stage 2 comprised 5 min of rapid marching or jogging at 8 km·h^-1^ and 0% grade. Thereafter, speed remained at 8 km·h^-1^ and grade increased by 2 percentage points every 3 min until volitional exhaustion (stage 3: 2% grade; stage 4: 4% grade; and so forth). The stage variable refers solely to the treadmill speed and grade setting and does not represent an equivalent external task demand between conditions because total transported mass differed.

HR, V̇O_2_, carbon dioxide production (V̇CO_2_), minute ventilation (V̇E), and SmO_2_ were recorded continuously. Mean physiological values from the final 30 s at rest and of each 5- or 3-min stage were used for stage-level analyses. Participants completed the same protocol twice in randomized crossover order, with 2–14 days between tests, beginning with either NL or CB. NL consisted of body mass only, with running shoes, a top, and shorts or tights. CB consisted of body mass plus personal combat gear, armored plates, and a dummy weapon simulating the Swedish Army AK5. The soldiers were asked to bring their personal combat gear, which they were familiar with and fitted to their body size and position; thus, external load was individualized rather than standardized to body mass: 16.34 kg ± 2.51 kg in women and 15.70 kg ± 2.04 kg in men, corresponding to 24.84% ± 4.56% and 20.08% ± 1.65% of NL body mass, respectively. Cardiopulmonary variables were measured with an Oxycon Pro system (Jaeger, Hoechberg, Germany) mixing chamber, and the gas analyzers were calibrated immediately before each test in accordance with the manufacturer’s instructions. HR was measured with a Polar FT4 monitor (Polar Electro Oy, Kempele, Finland) synchronized with the Oxycon Pro system.

### Ventilatory thresholds

Ventilatory transitions were determined from gas-exchange and ventilatory responses obtained during the GXT (Wasserman, 1987; Galán-Rioja et al., 2020) from stage-aggregated data. VT1 was identified using the V-slope method as the breakpoint in the relation between V̇CO_2_ and V̇O_2_ (Schneider et al., 1993). VT2/RCP was identified visually from a secondary increase in V̇E/V̇O_2_ together with a marked increase in V̇E/V̇CO_2_, consistent with respiratory compensation (Wasserman, 1987; Keir et al., 2024). These ventilatory transitions were treated as surrogate markers associated with exercise-intensity-domain boundaries, not as direct or necessarily identical measures of the underlying metabolic boundaries. One investigator, blinded to sex and load condition, identified both transitions. No second assessor, consensus procedure, or formal intra-rater reliability assessment was available; therefore, this is acknowledged as a limitation. The percentage of condition-specific V̇O_2_peak at each threshold was calculated as (threshold V̇O_2_ divided by V̇O_2_peak from the same GXT condition) × 100.

### Muscle oxygenation

Muscle oxygenation was assessed non-invasively using NIRS. SmO_2_ was monitored with a Moxy Monitor (Fortiori Design LLC, MN, USA) placed over the right vastus lateralis midway between the greater trochanter and lateral femoral epicondyle at the point of greatest muscle girth, in accordance with previously described procedures (Hesford et al., 2012). The site was identified by palpation and marked to improve placement consistency between conditions. The sensor was secured with adhesive tape and an elastic covering to reduce movement artifact and ambient-light exposure. All NIRS outcomes therefore represent only the sampled region of the right vastus lateralis and should not be generalized to whole-body or whole-limb muscle oxygenation.

Standing SmO_2_ (SmO_2_baseline) was calculated as the average of a 30-s period after a steady value had been reached and was used as a reference, not as physiological normalization between participants (Hesford et al., 2013). SmO_2_ was extracted at VT1 and VT2, and the temporal minimum observed during the test was defined as SmO_2lowest_. The minimum was not assumed to coincide exactly with whole-body V̇O_2peak_. Baseline-referenced desaturation amplitude (ΔSmO_2_) was calculated as SmO_2baseline_ − SmO_2lowest_, so desaturation is denoted by a negative value. The SmO_2_ desaturation rate (%·min^-1^) likewise described the reduction from the standing reference to the temporal minimum and was reported as negative. Baseline subtraction does not remove the influence of adipose-tissue thickness or other optical differences on the NIRS signal.

### Recovery

Post-exercise responses were assessed using one central and one local index. Heart-rate recovery at 1 min (HRR1min) was calculated as HR at exercise cessation minus HR after 1 min (Petek et al., 2023). The early SmO_2_ resaturation slope was calculated as the increase in right vastus lateralis SmO_2_ during the first 10 s after exercise cessation, with the participants sitting down on a chair, divided by 10 and expressed in percentage points·s^-1^. It was treated as an exploratory index of early local reoxygenation/reperfusion rather than recovery in general.

### Statistical analyses

Data are presented as mean ± standard deviation (SD). Analyses were performed using SPSS Statistics version 28 (IBM Corporation, NY, USA), with α <0.05. Independent-samples t-tests compared body composition and external load between sexes. Welch’s correction was used when homogeneity of variance was violated. For V̇O_2_, HR, RER, HRR, and SmO_2_ outcomes at VT1, VT2, peak effort, and recovery, a 2 (load: NL/CB; within-subject) × 2 (sex: women/men; between-subject) mixed ANOVA was used. The SmO_2_ profile was examined using a 4 (time: baseline, VT1, VT2, temporal minimum) × 2 (load) × 2 (sex) mixed ANOVA. Model residuals were evaluated using Q–Q plots and Shapiro–Wilk tests, homogeneity of variance for between-subject terms was assessed with Levene’s test, and standardized residuals with an absolute value >3 were flagged as potential outliers. Sphericity is automatically satisfied for the two-level load factor. For effects involving the four-level time factor, sphericity was assessed using Mauchly’s test, and Greenhouse–Geisser correction was applied when violated. All load, sex, and interaction terms are reported with F statistics, degrees of freedom, exact p values, and partial eta squared (ηp²). Effect estimates are reported with two-sided 95% confidence intervals. Within-sex NL–CB effect sizes were calculated as Cohen’s *d_av_* for the within-participant comparison between CB and NL conditions. In this study, positive *d_av_* values denote higher values in the CB condition; Cohen’s *d_s_* was used to quantify between-sex differences. It was calculated as the difference between the mean value for men and the mean value for women, divided by the pooled SD (Cohen, 1988, Hopkins et al., 2009, Lakens, 2013). For sex contrasts averaged across load, the group means and pooled SD were calculated from each participant’s mean of the NL and CB values. Moreover, positive *d_s_* values denote higher values in men. A significant contrast in one sex and a non-significant contrast in the other were not interpreted as a sex-specific response without a supporting interaction. Non-significant tests were considered inconclusive and not evidence of similarity or equivalence. Analyses involving sex and sex-by-load interactions were exploratory because of the small sample.

## Results

Women had lower body mass and FFM and higher total and right-leg fat percentages than men, whereas the groups had the same mean BMI (**Table 1**). Absolute external load did not differ between sexes (mean difference women minus men 0.64 kg, 95% CI −1.64 to 2.93; t(16) = 0.60, p = 0.559, d_s_ = −0.28). However, relative external load was 4.77 percentage points greater in women (95% CI 1.17 to 8.36; Welch t(10.05) = 2.95, p = 0.014; d_s_ = −1.39 for men minus women) (**Table 1**). For context, previously reported total test duration (time to volitional exhaustion) was 20.3 min ± 2.79 min in NL and 10.7 min ± 2.59 min in CB for men, and 20.1 min ± 3.81 min in NL and 9.0 min ± 2.56 min in CB for women (Larsson et al., 2022). Elapsed time at VT1 and VT2 was not retained as a separate outcome.

**Table 1:** Body composition and individualized external load of female and male soldiers (n = 18).

Subject characteristics	Women (n = 9)	Men (n = 9)	P value (sex comparison)	Cohen´s *d_s_*
Body mass (kg)	66.5 ± 8.6	78.3 ± 8.9	0.012	1.35
FFM (kg)	50.7 ± 5.4	68.9 ± 7.9	<0.001	2.69
FFM right leg (kg)	5.2 ± 0.7	9.3 ± 1.1	<0.001	4.45
Body fat (%)	23.6 ± 2.8	11.4 ± 4.5	<0.001	−3.26
Right leg fat (%)	31.4 ± 3.8	13.4 ± 4.7	<0.001	−4.21
BMI (kg/m2)	23.7 ± 2.2	23.7 ± 2.2	0.950	0.00
Absolute external load (kg)	16.3 ± 2.5	15.7 ± 2.0	0.559	−0.28
External load (% of NL body mass)	24.8 ± 4.6	20.1 ± 1.6	0.014	−1.39

Values are mean ± SD. p values are for independent between-sex comparisons; †Welch’s t-test was used because homogeneity of variance was.

violated. Cohen’s *d_s_* is positive when men had the higher value. BMI, body mass index; FFM, fat-free mass. Armor plates and combat equipment.

were individually fitted, producing variation in absolute and relative load.

### Peak oxygen consumption and load carriage

Peak responses from this experiment have been published previously (Larsson et al., 2022) and are presented here only to contextualize the threshold analyses. Absolute V̇O_2peak_ decreased from NL to CB by 11% (ES = −0.94) in men and 5.6% in women (ES = −0.55) (**Table 2**). The reported mL·kg^-1^·min^-1^ variable uses body mass as the denominator in NL but total transported mass (body mass plus CB) in CB; it is therefore a transported-system-mass-normalized value and its 24% reduction should not be interpreted as a 24% deterioration in aerobic capacity. V̇O_2peak_ relative to FFM was also lower in CB. The available tests did not provide evidence of load or sex effects on peak RER (**Table 2**); this does not establish equivalent effort between conditions or sexes.

### Ventilatory thresholds and load carriage

Treadmill stage, HR, absolute V̇O_2_, and percentage of condition-specific V̇O_2peak_ (%VO_2peak_) were examined at VT1 and VT2. Complete load, sex, and load × sex statistics, marginal estimates with 95% CIs, and within-sex paired estimates are reported in **Table 2**.

### Ventilatory threshold 1

VT1 was identified at a lower treadmill stage in CB than in NL (main effect of load, p <0.01) (women ES = −1.75; men ES = −0.90); exploratory within-sex contrasts were p <0.01 in women and p = 0.02 in men. Absolute V̇O_2_ at VT1 changed little between the NL and CB conditions (men: 2.78 to 2.72 L·min^-1^; women: 2.24 to 2.22 L·min^-1^), whereas HR was higher in CB (main effect of load, p <0.001; **Table 2**) (women ES = 0.72, men ES = 1.10). These findings indicate that a similar absolute V̇O_2_ was reached at a lower treadmill setting while participants transported more mass and had a higher cardiovascular response. Percentage of V̇O_2peak_ at VT1 showed a load effect (p = 0.02, women ES = 0.51, men ES = 1.10); the exploratory contrasts were p = 0.02 in men and p = 0.20 in women. However, this contrast pattern alone does not demonstrate a male-specific response.

### Ventilatory threshold 2

VT2 was also identified at a lower treadmill stage in CB than in NL (main effect of load, p <0.01; exploratory within-sex contrasts p <0.01 in both groups) (women ES = −2.73, men ES = −3.13). Absolute V̇O_2_ at VT2 was approximately 5% lower in CB in both sexes, with reported main effects of load (p = 0.02) and sex (p <0.01; **Table 2**). The exploratory within-sex contrasts were p = 0.04 in men and p = 0.21 in women and should not be interpreted as evidence of a male-specific load effect without the corresponding interaction. No statistically significant effects were detected for percentage of V̇O_2peak_ or HR at VT2 (**Table 2**); given the sample size and absence of equivalence testing, these findings remain imprecise rather than demonstrating similarity.

### Regional muscle oxygenation

The right vastus lateralis SmO_2_ profile showed a time × sex interaction (p = 0.01). Exploratory simple contrast comparisons indicated less baseline-referenced desaturation in women than men at VT1, VT2, and the temporal minimum in both conditions (p <0.05; **Figure 1**). These are site-specific NIRS observations and may be influenced by the marked between-sex difference in right-leg fat percentage.

**Figure 1:**
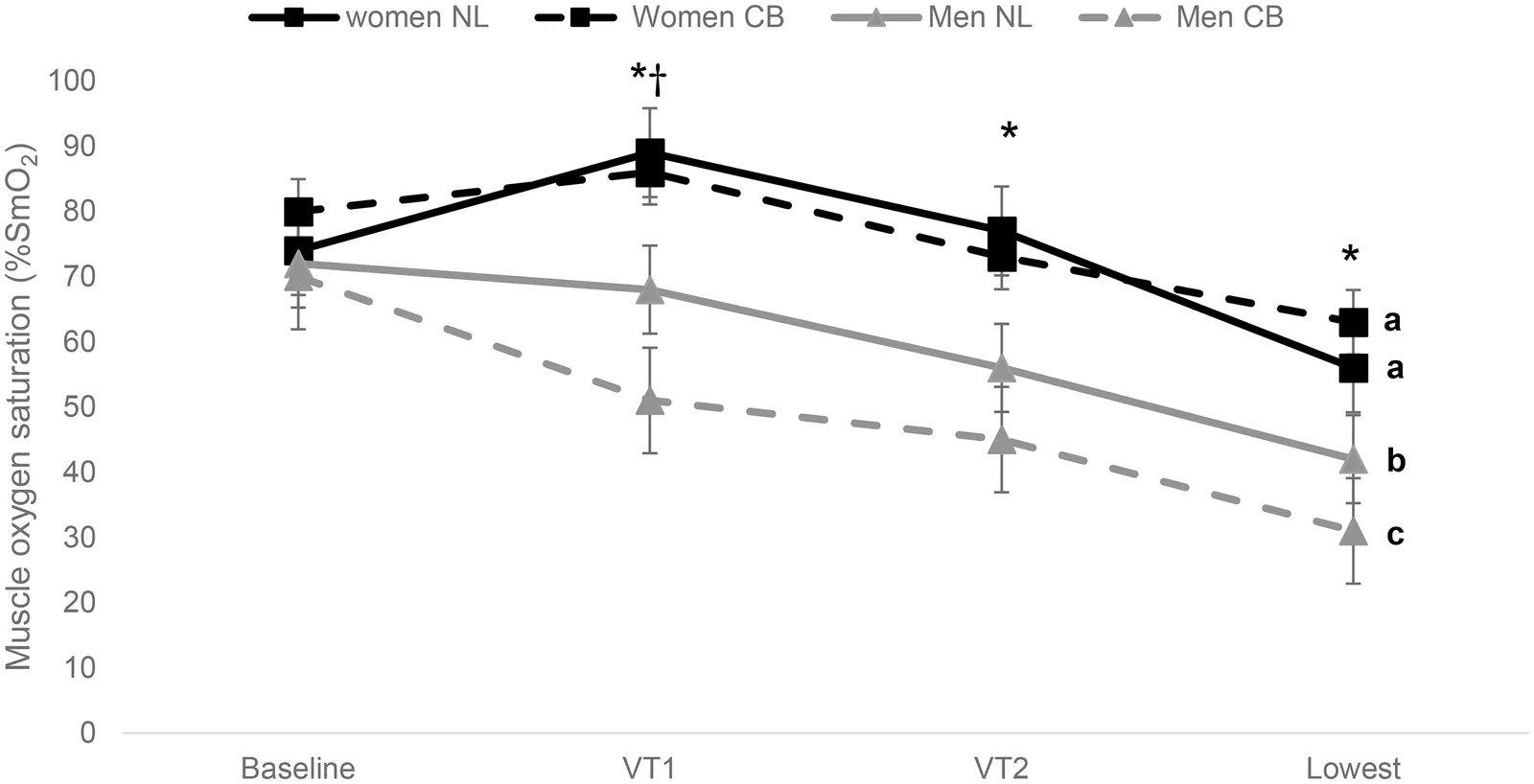
Right vastus lateralis muscle oxygen saturation (SmO_2_) from the standing reference to VT1, VT2, and the temporal minimum observed during the GXT under no-load (NL) and combat gear/body armor (CB) conditions. Squares represent women and triangles represent men; solid and dashed lines represent NL and CB, respectively. Values are site-specific, and baseline referencing does not correct for adipose-tissue thickness. *Exploratory *post-hoc* between-sex contrast at p <0.05. †Exploratory *post hoc* load contrast in men at p <0.05. Lettered annotations denote the reported *post-hoc* time-point contrasts.

Baseline-referenced desaturation amplitude (ΔSmO_2_) and desaturation rate were calculated from the standing reference to the temporal minimum. A sex main effect was reported for ΔSmO_2_ (p <0.01), with an exploratory between-sex contrast in CB of p <0.01 and in NL of p = 0.11 (**Table 2**).

### Recovery

No statistically significant load, sex, or load × sex effects were reported for HRR1min or the exploratory 10-s SmO_2_ resaturation slope (**Table 2**). Because the sample was small, these results do not demonstrate equivalent recovery between conditions or sexes.

Table 2Descriptive values, paired NL–CB estimates, effect sizes, and complete mixed-ANOVA results for women (W) and men (M).

**Table:** Panel A. Descriptive values and exploratory paired NL–CB contrasts.

Outcome	Sex	NL mean ± SD	CB mean ± SD	CB−NL [95% CI]	p_adj	*d_av_*
Peak values						
V̇O_2peak_ (L·min⁻¹)	M	4.16 ± 0.47	3.70 ± 0.51	-0.46 [-0.69, -0.23]	.004	-0.94
V̇O_2peak_ (L·min⁻¹)	W	3.23 ± 0.39	3.04 ± 0.33	-0.19 [-0.35, -0.03]	.052	-0.53
V̇O_2peak_ relative to transported mass (mL·kg⁻¹·min⁻¹)	M	53.34 ± 3.92	40.56 ± 3.53	-12.78 [-15.14, -10.42]	<.001	-3.43
V̇O_2peak_ relative to transported mass (mL·kg⁻¹·min⁻¹)	W	48.86 ± 5.83	36.92 ± 4.12	-11.94 [-14.70, -9.17]	<.001	-2.40
V̇O_2peak_ (mL·kg FFM⁻¹·min⁻¹)	M	60.65 ± 5.05	53.84 ± 5.15	-6.80 [-10.45, -3.16]	.005	-1.34
V̇O_2peak_ (mL·kg FFM⁻¹·min⁻¹)	W	63.84 ± 6.84	60.13 ± 5.88	-3.71 [-6.92, -0.51]	.057	-0.58
HRmax (beats·min⁻¹)	M	195.6 ± 8.7	194.9 ± 7.4	-0.7 [-5.4, 4.1]	1.000	-0.08
HRmax (beats·min⁻¹)	W	197.6 ± 7.6	196.4 ± 6.1	-1.1 [-3.5, 1.3]	.627	-0.16
RER at peak	M	1.10 ± 0.03	1.10 ± 0.04	+0.00 [-0.03, 0.03]	1.000	-0.03
RER at peak	W	1.10 ± 0.02	1.09 ± 0.04	-0.02 [-0.04, 0.01]	.480	-0.50
Ventilatory threshold 1 (VT1)						
GXT stage at VT1	M	6.4 ± 1.7	5.2 ± 0.8	-1.2 [-2.2, -0.2]	.045	-0.98
GXT stage at VT1	W	7.1 ± 1.5	5.1 ± 0.6	-2.0 [-3.2, -0.8]	.011	-1.95
V̇O₂ at VT1 (L·min⁻¹)	M	2.78 ± 0.50	2.73 ± 0.62	-0.05 [-0.31, 0.20]	1.000	-0.09
V̇O₂ at VT1 (L·min⁻¹)	W	2.24 ± 0.19	2.22 ± 0.09	-0.02 [-0.16, 0.12]	1.000	-0.15
Condition-specific V̇O2peak at VT1 (%)	M	66.4 ± 6.4	73.2 ± 9.1	+6.8 [-0.0, 13.5]	.101	0.87
Condition-specific V̇O2peak at VT1 (%)	W	70.0 ± 7.8	73.6 ± 6.1	+3.6 [-2.0, 9.2]	.347	0.52
HR at VT1 (beats·min⁻¹)	M	168.8 ± 13.3	180.3 ± 6.5	+11.6 [3.7, 19.4]	.019	1.17
HR at VT1 (beats·min⁻¹)	W	175.9 ± 9.8	183.6 ± 10.8	+7.7 [0.4, 14.9]	.083	0.75
Ventilatory threshold 2 (VT2)						
GXT stage at VT2	M	9.3 ± 0.7	6.6 ± 1.0	-2.8 [-3.7, -1.9]	<.001	-3.23
GXT stage at VT2	W	9.3 ± 1.1	6.3 ± 1.1	-3.0 [-3.4, -2.6]	<.001	-2.68
V̇O₂ at VT2 (L·min⁻¹)	M	3.49 ± 0.44	3.30 ± 0.47	-0.20 [-0.45, 0.06]	.232	-0.43
V̇O₂ at VT2 (L·min⁻¹)	W	2.78 ± 0.36	2.66 ± 0.29	-0.11 [-0.23, 0.00]	.108	-0.34
Condition-specific V̇O2peak at VT2 (%)	M	84.0 ± 6.6	89.5 ± 9.2	+5.5 [-2.0, 13.0]	.263	0.69
Condition-specific V̇O2peak at VT2 (%)	W	86.0 ± 3.5	87.8 ± 4.1	+1.8 [-1.9, 5.4]	.598	0.46
HR at VT2 (beats·min⁻¹)	M	187.4 ± 10.3	190.1 ± 7.0	+2.7 [-3.8, 9.1]	.734	0.31
HR at VT2 (beats·min⁻¹)	W	192.4 ± 9.3	193.1 ± 9.4	+0.7 [-3.5, 4.9]	1.000	0.07
Muscle oxygen saturation (SmO₂)						
Signed ΔSmO₂ (percentage points)	M	-30.8 ± 17.6	-39.0 ± 23.9	-8.2 [-34.6, 18.1]	.985	-0.40
Signed ΔSmO₂ (percentage points)	W	-17.8 ± 14.7	-17.1 ± 14.5	+0.7 [-14.9, 16.2]	1.000	0.05
Signed SmO₂ change rate (percentage points·min⁻¹)	M	-0.87 ± 0.55	-1.39 ± 0.83	-0.52 [-1.46, 0.42]	.483	-0.74
Signed SmO₂ change rate (percentage points·min⁻¹)	W	-0.49 ± 0.39	-0.69 ± 0.61	-0.20 [-0.75, 0.35]	.858	-0.40
Recovery						
HRR1min (beats·min⁻¹)	M	31.9 ± 11.8	30.0 ± 14.0	-1.9 [-11.0, 7.2]	1.000	-0.15
HRR1min (beats·min⁻¹)	W	27.0 ± 10.2	24.7 ± 7.8	-2.3 [-8.6, 3.9]	.831	-0.26
Early SmO₂ resaturation slope (percentage points·s⁻¹)	M	1.48 ± 1.15	0.83 ± 0.69	-0.64 [-1.37, 0.08]	.150	-0.70
Early SmO₂ resaturation slope (percentage points·s⁻¹)	W	0.53 ± 0.55	0.56 ± 0.48	+0.02 [-0.37, 0.41]	1.000	0.04

**Table:** Panel B. Complete 2 × 2 mixed-ANOVA results

Outcome	Load	Sex	Load × sex
V̇O_2peak_ (L·min⁻¹)	Est. -0.33 [-0.46, -0.20] F(1,16)=28.35; p<.001; ηp²=0.639	Est. +0.80 [0.39, 1.21] F(1,16)=17.16; p<.001; ηp²=0.517	Est. -0.27 [-0.53, -0.01] F(1,16)=4.83; p=.043; ηp²=0.232
V̇O_2peak_ relative to transported mass (mL·kg⁻¹·min⁻¹)	Est. -12.36 [-14.03, -10.69] F(1,16)=246.11; p<.001; ηp²=0.939	Est. +4.06 [-0.05, 8.16] F(1,16)=4.39; p=.052; ηp²=0.215	Est. -0.84 [-4.18, 2.50] F(1,16)=0.29; p=.599; ηp²=0.018
V̇O_2peak_ (mL·kg FFM⁻¹·min⁻¹)	Est. -5.26 [-7.49, -3.03] F(1,16)=24.96; p<.001; ηp²=0.609	Est. -4.74 [-10.06, 0.58] F(1,16)=3.57; p=.077; ηp²=0.182	Est. -3.09 [-7.56, 1.37] F(1,16)=2.16; p=.161; ηp²=0.119
HRmax (beats·min⁻¹)	Est. -0.9 [-3.3, 1.5] F(1,16)=0.60; p=.450; ηp²=0.036	Est. -1.8 [-8.8, 5.3] F(1,16)=0.28; p=.601; ηp²=0.017	Est. +0.4 [-4.4, 5.3] F(1,16)=0.04; p=.849; ηp²=0.002
RER at peak	Est. -0.01 [-0.03, 0.01] F(1,16)=0.80; p=.385; ηp²=0.047	Est. +0.00 [-0.02, 0.03] F(1,16)=0.08; p=.776; ηp²=0.005	Est. +0.01 [-0.03, 0.05] F(1,16)=0.60; p=.451; ηp²=0.036
GXT stage at VT1	Est. -1.6 [-2.3, -0.9] F(1,16)=22.28; p<.001; ηp²=0.582	Est. -0.3 [-1.3, 0.7] F(1,16)=0.36; p=.556; ηp²=0.022	Est. +0.8 [-0.7, 2.2] F(1,16)=1.30; p=.271; ηp²=0.075
V̇O₂ at VT1 (L·min⁻¹)	Est. -0.04 [-0.17, 0.10] F(1,16)=0.33; p=.575; ηp²=0.020	Est. +0.52 [0.13, 0.91] F(1,16)=8.11; p=.012; ηp²=0.336	Est. -0.03 [-0.30, 0.24] F(1,16)=0.07; p=.802; ηp²=0.004
Condition-specific V̇O2peak at VT1 (%)	Est. +5.2 [1.2, 9.2] F(1,16)=7.43; p=.015; ηp²=0.317	Est. -2.0 [-8.3, 4.2] F(1,16)=0.48; p=.499; ηp²=0.029	Est. +3.1 [-4.9, 11.2] F(1,16)=0.69; p=.420; ηp²=0.041
HR at VT1 (beats·min⁻¹)	Est. +9.6 [4.7, 14.5] F(1,16)=17.07; p<.001; ηp²=0.516	Est. -5.2 [-14.3, 4.0] F(1,16)=1.44; p=.247; ηp²=0.083	Est. +3.9 [-6.0, 13.8] F(1,16)=0.70; p=.415; ηp²=0.042
GXT stage at VT2	Est. -2.9 [-3.3, -2.4] F(1,16)=177.31; p<.001; ηp²=0.917	Est. +0.1 [-0.8, 1.0] F(1,16)=0.07; p=.795; ηp²=0.004	Est. +0.2 [-0.7, 1.1] F(1,16)=0.26; p=.616; ηp²=0.016
V̇O₂ at VT2 (L·min⁻¹)	Est. -0.15 [-0.28, -0.03] F(1,16)=6.44; p=.022; ηp²=0.287	Est. +0.67 [0.30, 1.05] F(1,16)=14.38; p=.002; ηp²=0.473	Est. -0.08 [-0.34, 0.18] F(1,16)=0.46; p=.509; ηp²=0.028
Condition-specific V̇O_2peak_ at VT2 (%)	Est. +3.6 [-0.2, 7.5] F(1,16)=3.98; p=.063; ηp²=0.199	Est. -0.2 [-5.2, 4.8] F(1,16)=0.01; p=.933; ηp²=0.000	Est. +3.7 [-4.0, 11.4] F(1,16)=1.06; p=.319; ηp²=0.062
HR at VT2 (beats·min⁻¹)	Est. +1.7 [-1.9, 5.2] F(1,16)=1.00; p=.332; ηp²=0.059	Est. -4.0 [-12.4, 4.4] F(1,16)=1.03; p=.326; ηp²=0.060	Est. +2.0 [-5.1, 9.1] F(1,16)=0.36; p=.557; ηp²=0.022
Signed ΔSmO₂ (percentage points)	Est. -3.8 [-17.8, 10.3] F(1,16)=0.32; p=.577; ηp²=0.020	Est. -17.4 [-28.8, -6.1] F(1,16)=10.67; p=.005; ηp²=0.400	Est. -8.9 [-37.0, 19.2] F(1,16)=0.45; p=.513; ηp²=0.027
Signed SmO₂ change rate (percentage points·min⁻¹)	Est. -0.36 [-0.86, 0.14] F(1,16)=2.29; p=.150; ηp²=0.125	Est. -0.54 [-0.90, -0.18] F(1,16)=10.32; p=.005; ηp²=0.392	Est. -0.32 [-1.32, 0.69] F(1,16)=0.44; p=.514; ηp²=0.027
HRR1min (beats·min⁻¹)	Est. -2.1 [-7.2, 3.0] F(1,16)=0.78; p=.390; ηp²=0.046	Est. +5.1 [-4.9, 15.1] F(1,16)=1.18; p=.293; ηp²=0.069	Est. +0.4 [-9.7, 10.6] F(1,16)=0.01; p=.927; ηp²=0.001
Early SmO₂ resaturation slope (percentage points·s⁻¹)	Est. -0.31 [-0.69, 0.07] F(1,16)=3.02; p=.101; ηp²=0.159	Est. +0.61 [-0.05, 1.27] F(1,16)=3.83; p=.068; ηp²=0.193	Est. -0.67 [-1.43, 0.09] F(1,16)=3.47; p=.081; ηp²=0.178

**Table:** Panel C. Complete 4 (time) × 2 (load) × 2 (sex) SmO₂-profile model.

Effect	Estimate [95% CI]	F (df)	p; ηp²
Load	-5.57 [-12.46, 1.32]	2.94 (1, 16)	.106; 0.155
Sex	-21.49 [-36.92, -6.05]	8.70 (1, 16)	.009; 0.352
Load × sex	-6.86 [-20.63, 6.91]	1.12 (1, 16)	.307; 0.065
Time	—	18.57 (1.97, 31.48)	<.001; 0.537
Time × sex	—	6.73 (1.97, 31.48)	.004; 0.296
Time × load	—	1.92 (1.59, 25.42)	.174; 0.107
Time × load × sex	—	0.61 (1.59, 25.42)	.513; 0.037

Values are mean ± SD. In Panel A, differences are CB−NL with two-sided 95% CIs; p_adj values are Bonferroni-adjusted across the two exploratory within-sex contrasts. d_av_ = (M_CB−M_NL)/[(SD_CB+SD_NL)/2]. These contrasts do not establish sex-specific responses without a significant interaction. In Panel B, load estimates are marginal CB−NL differences, sex estimates are men−women averaged across load, and interaction estimates are (CB−NL)_men−(CB−NL)_women. Panel C reports Greenhouse–Geisser-corrected time-related tests. V̇O₂, oxygen uptake; FFM, fat-free mass; HR, heart rate; RER, respiratory exchange ratio; GXT, graded exercise test; HRR1min, 1-min heart-rate recovery; ΔSmO₂, signed baseline-referenced change. Transported-mass V̇O2peak uses body mass in NL and body mass plus CB in CB. p_adj, Bonferroni-adjusted p value for the within-sex NL–CB comparison.

## Discussion

This study examined how CB changed the relationship between treadmill setting and internal physiological responses at ventilatory transitions, together with site-specific right vastus lateralis SmO_2_ and exploratory early post-exercise indices. CB was associated with VT1 and VT2 being identified at lower treadmill stages, but this should not be interpreted as proof that the underlying metabolic thresholds shifted. At VT1, absolute V̇O_2_ changed little while HR was higher, indicating a changed relationship between the treadmill setting, transported mass, and cardiovascular response. The SmO_2_ findings were local and baseline-referenced, and the exploratory recovery analyses were inconclusive. Sex-related NIRS patterns require particular caution because the groups differed markedly in right-leg fat percentage and the sample was small.

### Peak oxygen consumption and load carriage

Peak V̇O_2_ responses from this experiment were reported previously (Larsson et al., 2022) and are not presented as a novel finding here. Absolute V̇O_2_peak was lower in CB, and the significant load × sex interaction indicated a larger reduction in men than women. The 24% decrease in the transported-mass-normalized mL·kg^-1^·min^-1^ values is partly imposed by using body mass in NL and body mass plus CB in the loaded denominator and describes oxygen uptake relative to total transported system mass rather than a 24% loss of aerobic capacity. A body-mass-normalized analysis using the same denominator definition in both conditions is required for a direct physiological comparison. Differences of only a few percent should also be considered in relation to measurement and biological variability. Recent simulator work found that Oxycon Pro accuracy depends on configuration and reported a mean V̇O_2_ error of 2.72% ± 2.38% and technological variability of approximately 0.46% for the tested mixing-chamber configuration (Van Hooren et al., 2026). In athletes, Knaier et al. (2019) reported day-to-day and within-person diurnal V̇O_2max_ differences of 2.0 mL·kg^-1^·min^-1^ ± 1.0 mL·kg^-1^·min^-1^ and 5.0 mL·kg^-1^·min^-1^ ± 1.9 mL·kg^-1^·min^-1^, respectively. The Oxycon Pro operating mode, calibration details, and test timing are therefore important when interpreting the smaller observed differences.

### Ventilatory thresholds and load carriage

The most consistent finding was that both ventilatory transitions were identified at lower treadmill stages during CB. These stage changes were 19% in men and 28% in women at VT1 and 29% and 32%, respectively, at VT2. It is important to remember that these are percentage changes in ordinal stage number, not percentage reductions in treadmill grade. Neither stage (VT1/VT2) showed a load × sex interaction. At VT1, there was no load × sex interaction, while HR increased. Thus, the data support an altered relationship between the task setting and internal cardiovascular strain, but the data do not demonstrate displacement of the underlying metabolic threshold. Additional mass, possible thoracic restriction, altered gait and movement mechanics, weapon carriage, and local fatigue are all plausible contributors; however, the present design cannot separate them. Women carried a significantly greater relative load than men, which further limits causal interpretation of sex comparisons. Phillips et al. (2016) similarly showed that external loading alters the relationship between locomotor work rate and internal responses.

At VT2, absolute V̇O_2_ was approximately 5% lower in CB in both groups, whereas the analyses did not detect effects on percentage of V̇O_2peak_ or HR. These non-significant findings should not be interpreted as equivalence. In a recent randomized comparison, a vest-loaded incline-ramp protocol and an unloaded speed-ramp protocol produced practically comparable V̇O_2peak_ and threshold responses despite substantially different treadmill settings (Dössegger et al., 2026). Together with the present data, this emphasizes that treadmill setting, total transported mass, and internal physiological transition markers should be reported separately. Protocol design is also relevant: the two initial 5-min stages and subsequent 3-min stages provide relatively coarse stage-based resolution, and thresholds occurring at different times within a stage receive the same ordinal stage value.

Taken together, CB changed the treadmill setting at which the ventilatory transitions were observed and increased HR at VT1 without materially changing absolute V̇O_2_ at VT1. This result supports task-specific loaded assessment when the aim is to characterize physiological strain during equipment carriage. It does not show that an unloaded physiological threshold itself moved, nor can it identify which component of the CB ensemble caused the altered response.

### Muscle deoxygenation and load carriage

Right vastus lateralis SmO_2_ provided a local signal complementary to pulmonary gas exchange, but it should not be generalized to overall muscle oxygenation. The time × sex interaction was followed by adjusted contrasts showing lower SmO_2_ in men at VT1 and at the temporal minimum when averaged across load. Similar sex patterns have been reported during unloaded repeated-sprint and incremental-cycling exercise (Smith and Billaut, 2012; Espinosa-Ramirez et al., 2021). However, baseline subtraction does not remove optical effects of adipose tissue. In the present sample, right-leg fat was 31.4% ± 3.8% in women and 13.4% ± 4.7% in men, and site-specific adipose-tissue thickness was not measured. The apparent sex pattern may therefore reflect physiological differences, measurement-related differences, or both.

SmO_2lowest_ was the temporal minimum recorded in the right vastus lateralis and should not be equated automatically with whole-body maximal effort or the response of other locomotor muscles. Previous studies have reported heterogeneous sex differences in vastus lateralis oxygenation during incremental exercise (Bhambhani et al., 1998; Peltonen et al., 2013). Greater muscle mass, recruitment patterns, perfusion, and oxygen utilization are plausible physiological explanations, whereas adipose-tissue thickness and other optical characteristics are plausible measurement explanations. Because the present design measured one site and did not quantify adipose-tissue thickness at the sensor, these mechanisms cannot be separated.

### Recovery

Short intervals between repeated physical efforts are operationally relevant (Nindl et al., 2018), but the present measurements captured only HR change at 1 min and a 10-s right vastus lateralis resaturation slope. No statistically significant effects were detected. The 10-s SmO_2_ slope is particularly sensitive to posture, movement, and muscle-pump effects and is best regarded as an exploratory early local reoxygenation/reperfusion index. It should not be generalized to systemic or functional recovery.

### Limitations

Several limitations constrain interpretation. First, the sample was small, particularly for sex-by-load interactions, and sex-related analyses were exploratory. Second, the long staged protocol provides limited temporal resolution when ordinal stage at VT is used as an outcome; total elapsed time to each transition was not retained as a separate reported variable. Third, one blinded investigator identified VT1 and VT2 without a second assessor, consensus procedure, or formal intra-rater reliability assessment. Fourth, the available datasets did not contain the randomized test-sequence variable, so an order effect on V̇O_2peak_ could not be independently evaluated in the present reanalysis. Fifth, small V̇O_2_ differences should be interpreted in relation to the Oxycon Pro operating configuration and plausible measurement, day-to-day, and time-of-day variability (Knaier et al., 2019; Van Hooren et al., 2026). Sixth, NIRS sampled only the right vastus lateralis and is affected by adipose-tissue thickness (Van der Zwaard et al., 2016), yet skinfold thickness at the sensor site was not measured. Right-leg fat percentage was only an indirect estimate and cannot correct the SmO_2_ signal. Finally, the 10-s SmO_2_ slope was sensitive to movement and position, and recovery was assessed over too short an interval to represent broader physiological or functional recovery. These laboratory results may not generalize to prolonged field tasks or other load-carriage configurations.

## Conclusion

Combat gear and body armor was associated with VT1 and VT2 being identified at lower treadmill settings during the graded test. At VT1, the analysis did not detect a load effect on absolute V̇O_2_, whereas HR was higher, indicating a changed relationship between the treadmill task and internal cardiovascular response rather than demonstrated displacement of the underlying metabolic threshold. Right vastus lateralis SmO_2_ and early reoxygenation findings were site-specific and exploratory, with between-sex interpretation limited by sample size and unmeasured adipose-tissue thickness at the sensor. Loaded testing may improve task specificity, but the separate contributions of added mass, thoracic restriction, movement mechanics, weapon carriage, and local fatigue cannot be determined from this study.

## Data Availability

The raw data supporting the conclusions of this article will be made available by the authors, without undue reservation.
